# Childhood traumatization and dissociative experiences among maladaptive and normal daydreamers in a Hungarian sample

**DOI:** 10.1007/s12144-021-02223-3

**Published:** 2021-08-30

**Authors:** Alexandra Sándor, Antal Bugán, Attila Nagy, Nikolett Nagy, Katalin Tóth-Merza, Judit Molnár

**Affiliations:** 1 Department of Behavioural Sciences, Faculty of Medicine, University of Debrecen, Móricz Zsigmond krt. 22, Debrecen, H-4032 Hungary; 2 Doctoral School of Health Sciences, University of Debrecen, Debrecen, Hungary; 3 Faculty of Public Health, University of Debrecen, Kassai út 26, Debrecen, H-4028 Hungary; 4 Benedek Elek Faculty of Pedagogy, University of Sopron, Ferenczi J. u. 5, Sopron, H-9400 Hungary

**Keywords:** Maladaptive daydreaming, Childhood traumatization, Dissociative propensity, Hungarian sample

## Abstract

The aim of the study was to identify some potential etiological segments of maladaptive daydreaming, especially the relationships between maladaptive daydreaming, childhood traumatization, and dissociative propensity. The questionnaire package included the Hungarian version of the Maladaptive Daydreaming Scale, the Traumatic Antecedents Questionnaire, as well as the Dissociation Questionnaire. 717 participants were recruited online, 106 of whom were problematic daydreamers. The results revealed that certain types of childhood trauma occurred significantly more frequently in the group of maladaptive daydreamers. Furthermore, maladaptive daydreamers possessed a significantly higher level of dissociative propensity compared to normal daydreamers. The estimated SEM models showed that dissociative experiences - more precisely Identity confusion and fragmentation and Lack of control – mediated the relationship between certain childhood traumatic experiences and maladaptive daydreaming. The results suggest that we should consider childhood traumatization and increased dissociative propensity as potentially significant factors in the etiology of maladaptive daydreaming.

## Introduction

### Maladaptive Daydreaming

Daydreaming is a mental activity which enables attention to divert away from monitoring the external environment or performing actual tasks to observing the internal world (Singer, 2014). Daydreaming and fantasizing are universal human phenomena, which do not generate any difficulties for the majority of people. In fact, these mental phenomena have various adaptive functions: they promote the learning process, planning and thinking, stimulate creativity and the development of social skills, as well as help to cope with frustrations (Glausiusz, 2009; Killingsworth & Gilbert, 2010).

The pathological type of daydreaming, causing severe distress and functional impairment, was first described in 2002 (Somer, 2002). Maladaptive daydreaming is an excessive, pervasive, immersive and vivid fantasy activity, which results in a significant waste of time, loss of control and feelings of being addicted, as well as impairing daily functionality: i.e. forming and maintaining relationships, and/or academic and professional development (Somer & Herscu, 2017; Somer et al., 2016a). Maladaptive daydreaming is a proposed mental disorder characterised by intense yearning for being absorbed in vivid, detailed and eventful fantasies – often triggered or maintained by kinaesthetic activities and music (Soffer-Dudek et al., 2020).

Despite the severe negative consequences, the mental activity of maladaptive daydreaming can also play some subjectively rewarding roles in the daydreamers’ life and can function as self-entertainment, distraction from boredom and painful experiences and as a compensatory or wish fulfilment strategy (Somer, 2002; Somer et al., 2020b). Although there are some seemingly beneficial characteristics, maladaptive daydreaming cannot be considered an effective emotion regulation strategy (Sándor et al., 2021; Somer et al., 2020b; West & Somer, 2020). Previous studies explored that poorer emotion regulation ability was related to a higher degree of maladaptive daydreaming symptoms (Greene et al., 2020), and maladaptive and immersive components of the phenomenon were associated with poorer emotion regulation. These findings revealed that none of the forms of immersive daydreaming functions as an effective emotion regulation strategy (West & Somer, 2020). Further results showed that probable maladaptive daydreamers seemed to be less aware of their emotions, negative emotions might be overwhelming for them, and they also had difficulties with coping with these emotions and controlling their impulses (Sándor et al., 2021). Furthermore, individuals with probable maladaptive daydreaming, in response to self-isolation and quarantine (due to the COVID-19 lockdown restrictions), experienced more intensified and vivid fantasies, a stronger need to daydream and a higher level of psychological distress related to the pandemic: compared to the period before lockdown, these individuals reported more concentration problems, more worries about the future, boredom, loneliness, depression, social anxiety, more obsessions and compulsions, lower satisfaction with life, and decreased self-worth (Somer et al., 2020b). The conclusion of the study was that although maladaptive daydreaming originally could be a normal mental activity with entertaining and coping functions, the presence of major stressors might impair the psychosocial functionality (Somer et al., 2020b).

Somer et al. (2016a) developed the model of maladaptive daydreaming by describing the possible antecedents, features and maintaining factors, and the consequences of this excessive mental activity. According to the model, maladaptive daydreamers possess an innate capacity for vivid fantasy, and they tend to discover the rewarding characteristics of daydreaming in the early years of life. For many maladaptive daydreamers, the inner fantasy world functions as a source of security, calm and joy. These daydreamers often experience loneliness, lack of attention, friendlessness, and rejection in their childhood, which is in line with the results of the pioneering study of Somer (2002). The innate capacity for vivid fantasy, however, cannot lead to maladaptive daydreaming in itself, but other circumstances are required. The normal form of daydreaming develops into maladaptive when the inner absorption becomes irresistible and time consuming, the individual loses control over this mental behaviour, and therefore experiences severe distress and functional impairment (Somer, 2002; Somer et al., 2016a). Maladaptive daydreamers in the study (Somer et al., 2016a) describe the intense yearning for daydreaming as an addiction. It should be noted that the inborn capacity for daydreaming and the yearning for the rewarding experience of fantasizing further distance the individual from real life interactions resulting in a complex and circular interaction between the capacity for daydreaming and social isolation. The tendency to build a compensatory inner world and to avoid the painful reality seems to be even more significant among those individuals who were exposed to childhood abuse and/or neglect, or who were living in a dysfunctional family. For these individuals, daydreaming compensates for real life deficits and reduces stress and anxiety (Somer et al., 2016a). This result highlights the two main functions of maladaptive daydreaming previously described by Somer (2002, p.197), i.e. disengagement from stress and pain by mood enhancement and wish fulfillment fantasies, and companionship, intimacy, and soothing. For maladaptive daydreamers without a history of trauma, excessive fantasy activity provides pleasure and functions as a source of creativity (Somer et al., 2016a), which is consistent with Bigelsen and Schupak (2011) findings. Other factors which might be relevant in the maintenance of maladaptive daydreaming are the following: intense fear of being discovered; shame of being misunderstood; refusal to talk about daydreaming in therapy (Somer et al., 2016a), which results confirm the previous findings of Somer (2002). The tendency to hide their secretive behaviour from relatives and friends deprives maladaptive daydreamers from social support, and further increases the level of loneliness and distress. Moreover, when maladaptive daydreamers attempt to seek for professional help, they do not receive effective treatment due to the unfamiliarity of mental health professionals with the phenomenon and to the therapeutic approaches based on misdiagnosis (Somer, 2002; Somer et al., 2016a).

The seminal paper by Somer (2002) provoked intense reactions among individuals affected by the disorder, who have been living isolated lives, and been misunderstood, frequently misdiagnosed in the absence of adequate diagnosis (Bigelsen et al., 2016). Although these daydreamers tend to exchange information with each other, and provide peer support by creating websites, forums and online communities, the phenomenon has remained poorly understood among professionals. The international literature on maladaptive daydreaming consists of less than 55 published articles, while only one paper related to the topic has been published in Hungarian so far (Sándor & Molnár, 2018).

### Childhood Traumatization

International studies have revealed controversial findings regarding the relationship between childhood trauma and maladaptive daydreaming (Abu-Rayya et al., 2020; Bigelsen & Schupak, 2011; Bigelsen et al., 2016; Sándor et al., 2020; Somer, 2002; Somer et al., 2020a; Somer et al., 2019a; Somer & Herscu, 2017; Somer et al., 2016a). In his seminal paper, Somer (2002) described six patients with trauma history. In these participants’ cases, daydreaming started during childhood and functioned as a form of coping with trauma by distancing the individuals from adverse experiences. In a subsequent study, Somer and his colleagues (Somer et al., 2016a) confirmed that vivid fantasy activity might facilitate coping with adverse family circumstances in childhood. Participants in the study reported a stronger need to escape to the fantasy world if they had experienced traumatic childhood events or feelings of loneliness and isolation. In their model, authors described the hypothesis of a trauma origin for maladaptive daydreaming; however, they also concluded that pathological daydreaming could develop without the individual having struggled with childhood trauma (Somer et al., 2016a). In 2016, using a large sample (N = 447) Bigelsen and her colleagues found no significant difference between the groups of maladaptive daydreamers and controls regarding childhood traumatization. However, the authors did not exclude the possibility that severe traumatization could increase survivors’ motivation to escape into their inner world. Somer and Herscu (2017) concluded that childhood trauma was one of the potential pathways to maladaptive daydreaming, but absorption and fantasy addiction were essential conditions for the connection between childhood traumatization and pathological daydreaming. In a recent study, Somer et al. (2019a) explored the idea that patients who recovered from substance use disorder were more likely to report a history of childhood trauma, particularly emotional neglect, as well as dissociative experiences and pathological daydreaming, compared to controls. Their findings showed that the propensity for extreme daydreaming partially mediated the relationship between childhood neglect and later dissociative mechanisms. Recent research (Abu-Rayya et al., 2020) on maladaptive daydreaming examined women who were exposed to sexual abuse in their childhood; these women showed a significantly higher level of maladaptive daydreaming compared to the control group. This study confirmed the original hypothesis (Somer, 2002) that childhood traumatization might increase the risk of abnormal daydreaming, which may function as a coping strategy to deal with inescapable adverse circumstances.

A recent study (Somer et al., 2020a) has revealed that maladaptive daydreaming was positively correlated with physical abuse and neglect, and emotional abuse and neglect; however, these correlations were small. Those maladaptive daydreamers - screened by the Maladaptive Daydreaming Scale (Somer et al., 2017b) - who experienced childhood physical and emotional neglect and emotional abuse, utilized maladaptive daydreaming to distract themselves from painful memories and distress and to regulate their difficult emotions. These results suggest that maladaptive daydreaming has a post-abusive role in the individuals’ lives.

In a sample of Hungarian daydreaming-prone individuals (Sándor et al., 2020), a short, 10-item self-report questionnaire, ACE-10 (Adverse Childhood Experience Questionnaire- Finding Your ACE Score, Anda et al., 2010; translated into Hungarian by Anikó Ujhelyiné Nagy and Ildikó Kuritárné Szabó in 2015) was used to assess childhood emotional, physical and sexual abuse, emotional and physical neglect, and five types of household dysfunction (parental separation or divorce; witnessing domestic violence towards the mother or foster mother; familial alcohol abuse or other psychoactive substance abuse; familial mental illness or a suicide attempt; household incarceration) among individuals (N = 160) screened as maladaptive (n = 63) or normal daydreamers (n = 97). In this study a significant correlation was found between the first five trauma types and maladaptive daydreaming, while there were no significant correlations between the five types of household dysfunction and maladaptive daydreaming. These results suggest that further research and a more detailed and in-depth investigation of the trauma origin of the phenomenon is needed.

### Dissociation

Regarding the relationship between dissociative mechanisms and maladaptive daydreaming, seven international papers have been published (Bigelsen et al., 2016; Ferrante et al., 2020; Soffer-Dudek & Somer, 2018; Somer & Herscu, 2017; Somer et al., 2016c; Somer et al., 2017a; Somer et al., 2019b), and to the best of our knowledge, no scientific study has investigated this connection in Hungary. Previous studies found a significant connection between maladaptive daydreaming and specific dissociative phenomena. The statistical analysis revealed significant correlations between the scores of the 14-item Maladaptive Daydreaming Scale (MDS-14; Somer et al., 2016c) and the overall score of Dissociative Experiences Scale (DES, Bernstein & Putnam, 1986 cited in Somer et al., 2016c), as well as the Depersonalization, Amnesia and Absorption subscales. However, the strongest relationship was found with the Absorption subscale: excessive daydreamers were characterised by an intensive absorptive propensity to immerse themselves in their external and internal experiences (Bigelsen et al., 2016; Somer et al., 2016c). Somer and Herscu (2017) confirmed the above-mentioned findings, disclosing a significant relationship between maladaptive daydreaming and absorption measured by the Tellegen Absorption Scale (Tellegen & Atkinson, 1974). The authors suggested that absorption in fantasy, as well as fantasy addiction (fantasy dependence) mediated the relationship between childhood trauma and pathological daydreaming. Another study investigating the comorbidity between maladaptive daydreaming and other disorders, revealed that 12.8% of maladaptive daydreamers (n = 39) met the criteria for a dissociative disorder (Somer et al., 2017a). In terms of prevalence data, a research (Waller & Ross, 1997) estimated a prevalence of 3.3% of pathological dissociation in the general population of North America. European research (Spitzer et al., 2006) reported frequency rates of pathological dissociation between 0.3% and 1.8% in non-clinical samples, 5.4% in psychiatric inpatients, 4.8% in eating disordered patients, and 2.2% in psychosomatic outpatients. Based on the review article of Sar (2011), the prevalence of dissociative disorders in inpatient units varies between 4.3% and 40.8%, while in outpatient units this percentage varies between 12% and 29% (see Table 1 on p.2 in Sar, 2011). In nonclinical settings, the prevalence of dissociative disorders is between 1.7% and 18.3% (see Table 2 on p. 3 in Sar, 2011). The large variance is influenced by the fact that the findings involved in the review were obtained in diverse countries with applying samples of varying sizes, and with the use of different diagnostic or screening instruments. The author suggests that the lifetime prevalence of dissociative disorders seems to be around 10% in the community and in clinical populations (Sar, 2011).

A recent study, applying a longitudinal daily-diary method, revealed a temporal relationship between daydreaming and dissociation, suggesting that the more intense the maladaptive daydreaming activity was, the more intense the following dissociative symptoms were (Soffer-Dudek & Somer, 2018). In another study (Somer et al., 2019b), analysis of the pictorial representations and verbal descriptions of maladaptive daydreamers’ self and their maladaptive daydreaming experience, showed a dissociation between the vivid inner life and the painful external reality. Maladaptive daydreamers depicted their self and their reality as divided; they experienced duality between the emotionally satisfying, vivid, rich and pleasant daydreaming filled with life, and their dysfunctional existence in the dullness and emptiness of real life.

Another study (Ferrante et al., 2020) investigated the possible role of emotional trauma, dissociation and shame in maladaptive daydreaming. Multiple mediation analysis revealed that the relationship between emotional trauma and maladaptive daydreaming was mediated by dissociative absorption and shame. This study identified one possible developmental pathway of maladaptive daydreaming. Emotional trauma seemed to be an important segment determining the severity of maladaptive daydreaming, and this relationship was completely determined by the mediation of dissociation and shame.

### Potential Categorization of Maladaptive Daydreaming

Somer (2018) described maladaptive daydreaming as a multifaceted clinical phenomenon. One possible categorization of maladaptive daydreaming suggested that it might be a disorder of dissociative absorption. According to Somer, maladaptive daydreaming has dissociative properties, mainly characterized by a propensity for absorption. Maladaptive daydreaming is characterized by “compulsive detrimental absorption in fanciful fantasy” (Somer et al., 2019a, p.207). However, the characteristics of maladaptive daydreaming also showed similarities with behavioural addiction and compulsive disorders; in some cases, this problematic daydreaming activity evolves into dependence which can be described as a compulsion to daydream causing significant time loss and severe distress (Somer et al., 2019a). This fantasy activity is so rewarding that it causes a yearning for daydreaming, a loss of control over fantasizing, and a significant wasting of time, and also interferes with interpersonal, academic and professional functioning, as well as leading to significant distress, social withdrawal, and shame (Bigelsen & Schupak, 2011; Pietkiewicz et al., 2018; Somer, 2002; Somer, 2018). According to a study (Somer et al., 2017a), 53.9% of maladaptive daydreamers (N = 39) met the criteria for an obsessive-compulsive or related disorder, but this rate was not as high as the rate of comorbidity for other disorders, such as attention deficit hyperactivity disorder (ADHD; 76.9%), anxiety disorder (71.8%) or depressive disorder (66.7%). In a recent study, Salomon-Small and her colleagues (Salomon-Small et al., 2021) explored the relationship between maladaptive daydreaming and obsessive-compulsive spectrum symptoms (OCSS) and the potential factors mediating the relationship between these constructs. The results revealed that maladaptive daydreaming and OCSS were significantly correlated and the association between maladaptive daydreaming and OCSS was mediated by dissociation and impaired sense of control. According to their findings maladaptive daydreaming was moderately related to obsessions and compulsions (but showed stronger relations to obsessions). These results suggested that impaired control and intrusiveness of thoughts and mental experiences might underly the relationship between maladaptive daydreaming and OCSS.

Further theories suggest that problematic daydreaming might be linked to disturbances of attention. Based on a comorbidity study (Somer et al., 2017a), the most frequent comorbid disorder among maladaptive daydreamers was ADHD; 76.9% of the participants (N = 39) met the criteria of ADHD, predominantly the inattentive type. The researchers suggested that, based on the respondents’ description, the inattention of maladaptive daydreamers was caused by their highly absorptive fantasy activity - distracting the individuals from the external world interactions and tasks -, rather than by attention disorder (Somer et al., 2017a). The authors concluded that maladaptive daydreaming can be differentiated from normal daydreaming (in terms of content, quantity, subjective experience, degree of control over daydreaming, and interference with functioning), showing high levels of comorbidity, but cannot be explained by other existing disorders described in the Diagnostic and Statistical Manual of Mental Disorders (DSM–5) (Somer et al., 2019a; Somer et al., 2017a).

### The Present Study

Trauma history and dissociative propensity might be potentially important segments of the etiology and pathomechanism of maladaptive daydreaming, although the results of previous studies are not convincing. To the best of our knowledge no studies have investigated the relationship between maladaptive daydreaming, adverse childhood experiences and dissociation in Hungary. Based on the literature of maladaptive daydreaming the aim of the present study was threefold. The first objective was to clarify the connection between maladaptive daydreaming and childhood trauma: to explore the potential role of childhood trauma in the etiology of pathological daydreaming, and to reveal which type of trauma could function as a risk factor for excessive daydreaming. The present study explored childhood traumatic experiences in two developmental periods: 0–6 years and 7–12 years. As the impact of traumatic experiences on the individual’s psychiatric vulnerability and the brain development are influenced by the age when the trauma has been experienced, it seems necessary to explore, apart from the severity of trauma, the age when the traumatic event occurred (Park et al., 2020). Research on maladaptive daydreaming explored that maladaptive daydreamers have an innate capacity for vivid fantasy which might become an addictive mental activity in response to the exposure to childhood neglect, abuse, household dysfunctions or social isolation (Somer et al., 2016a). Later study revealed that childhood trauma is one of the potential pathways leading to the phenomenon (Somer & Herscu, 2017). It seems necessary to understand in which developmental period the trauma has affected the child in a way to develop a maladaptive daydreaming activity.

The second aim of the study was to explore maladaptive daydreamers’ dissociative propensity. The last aim was to investigate the relationship between childhood traumatization, dissociation and maladaptive daydreaming building complex (structural equation) models (SEM).

## Method

### Sampling Procedure

This study is part of a larger research; some demographic data from the larger survey were published before (Sándor et al., 2021). Recruitment of the study participants took two forms. 1) Our research team advertised a call for participants through online announcements with the snowball sampling method. Members of Facebook communities and groups dedicated to daydreaming and psychology could access the questionnaire package via an online link. Furthermore, members of a closed online group (called Excessive daydreaming- Maladaptive daydreaming) created by our research team in 2015 to share information about maladaptive daydreaming among members, and to announce invitations to participate in research, were invited. 2) The availability of the questionnaire package was shared with each Hungarian student of the University of Debrecen through the Neptun Unified Education System (the University’s online student administration platform), reaching 21.595 individuals.

Online data collection was justified by the fact that the researchers encountered difficulties when trying to reach this specific research target group for personal interviews. Previous studies also highlighted that maladaptive daydreamers feel profound shame about their behaviour, and are strongly motivated to conceal their activity even from their family members or friends. They are also frightened to talk about their excessive fantasy activity even within a trustful therapeutic relationship (Bigelsen & Schupak, 2011; Schimmenti et al., 2020; Somer et al., 2016a). The online research method seemed appropriate based on the results of previous international studies (Abu-Rayya et al., 2019; Bigelsen & Schupak, 2011; Bigelsen et al., 2016; Somer et al., 2016c; West & Somer, 2020), as this form of survey provides complete anonymity and volunteer answering, and facilitates open and honest response even to the more personal questions such as those related to traumatic experiences.

The research was conducted in line with the Helsinki Declaration and prior to data collection was approved by the Regional and Institutional Research Ethics Committee at the Clinical Center of the University of Debrecen and by the Medical Research Council. After providing participants with a complete description of the study, written informed consent was obtained from each of them. Study participation was anonymous, voluntary and involved one criterion for inclusion, that the participant had reached the age of 18.

### Measures

The first set of questions covered the *demographic data*: age, gender, marital status, education, and employment. Participants were asked about their self-identified maladaptive daydreamer status: based on the screening question for maladaptive daydreaming (Somer et al., 2017b, p.180), study participants could determine whether the definition of problematic daydreaming is true or false for them.

The Hungarian form of the *Maladaptive Daydreaming Scale* was used (MDS-16, Somer et al., 2017b; Sándor et al., 2020) to discriminate between normal and maladaptive daydreaming. The self-report screening questionnaire contains 16 items, which can be ranked on a Likert scale ranging from 0% to 100% with 10% intervals. The MDS-16 was adapted for Hungarian use by Sándor et al. (2020), and the cut-off score was determined at 60 percentiles, or 35 points (an average score lower or equal to 35 points is considered to be normal, an average score higher than 35 is considered to be screened as maladaptive daydreaming). Cronbach’s alpha was 0.9231 in the present study.

The *Traumatic Antecedents Questionnaire* (TAQ, Van der Kolk & Smyth, 2010; adapted for Hungarian use by Katalin Merza in 2012) was used to measure any traumatic childhood experiences. The 40-item self-report questionnaire assesses childhood experiences in four developmental periods (0–6; 7–12; 13–18 years and adulthood) on 10 scales: safety, competence, neglect, separation, emotional, physical and sexual abuse, witnessing, alcohol and drugs and other traumas. Participants could rate the severity or frequency of the traumatic experiences from 0 (never/not at all) to 3 (often or very much/very frequent) and could also indicate if they ‘don’t know’ the answer. The ‘don’t know’ answers were coded as missing responses.

In the present study, we aimed to analyse only childhood experiences (0–6 years and 7–12 years), and particularly adverse experiences. As we hypothesized that adverse childhood experiences might influence the development of maladaptive daydreaming, we did not examine experiences from 13 to 18 years and adulthood, as well as the dimensions of Safety and Competence. Only the eight subscales related to traumatic experiences were involved in the analysis. The Cronbach’s alpha of the scale (analysing the internal consistency of the first two developmental periods) was 0.9402.

To examine the dissociative experiences of the participants, we applied the *Hungarian version of the Dissociation Questionnaire* (DISQ-H, Vanderlinden et al., 1993; Varga et al., 1996), which assesses the experiences of identity confusion and fragmentation, loss of control over behaviour, thinking and emotions, amnesia and absorption. Items could be rated on a 5-point Likert scale (1- not at all; 5- extremely). The Cronbach’s alpha was 0.9691 in the present study.

### Statistical Analysis

The normality of the data was checked with the Shapiro-Wilk test. The Mann-Whitney U test was performed due to the non-normal distribution, to compare the childhood traumatic experiences of maladaptive and normal daydreamers in two developmental periods (0–6 years and 7–12 years). The Mann-Whitney U test was also used to investigate the differences between the two study groups regarding their dissociative propensity. Then, the univariate and multivariate normality of the data were tested with mvtest command. Due to the non-normal distribution of the data, the Kruskal- Wallis Test was applied to measure the influence of each trauma type on maladaptive daydreaming. Then, we applied the method of robust regression to measure the direct impacts of the childhood traumatic experiences (which significantly affect the phenomenon of maladaptive daydreaming based on the results of the Kruskal-Wallis Test) and the dissociative experiences on maladaptive daydreaming. We involved in the analyses the gender, the age groups (cut at 18 and 35 years) and the level of education as general confounding variables.

Then, we tested the direct impacts of the childhood traumatic experiences, dissociative experiences, and the general confounding variables on maladaptive daydreaming separately according to two developmental periods, i.e. 0–6 years and 7–12 years. The next step of the analysis aimed to reveal the direct and indirect relationships between the variables (childhood trauma, dissociation, and maladaptive daydreaming) with Structural Equation Modeling. The Structural Equation Model (SEM) requires the multivariate normality of indicators. As the indicators of the present study violated the assumption of univariate normality, and altogether violated the multivariate normality assumption as well, the SEM was built applying the Asymptotically Distribution-Free (ADF) estimation method. This approach does not require univariate and multivariate normality and is often used as an alternative when data are non-normal (Ory & Mokhtarian, 2010). If latent variables are not normally distributed, ADF might produce more efficient estimates than ML (Maximum Likelihood) or QML (Quasimaximum Likelihood) (STATA, 2017). We relied on the following fit indices to justify the acceptability of the model: CFI scores and TLI scores above 0.9 indicate acceptable fit (Marsh et al., 2004), RMSEA values below 0.08 and SRMR scores below 0.10 indicate acceptable fit (Schermelleh-Engel et al., 2003).

Intercooled Stata version 13.0 was used for the statistical analyses.

## Results

### Demographic Information

During the initial recruitment process, 243 participants filled in our questionnaire package from various Facebook communities and groups; 48 of them (19.75%) were screened as maladaptive daydreamers based on the cut-off score of the MDS-16-HU (Sándor et al., 2020). Participants’ ages ranged from 18 to 78 years (M = 36.43; SD = 12.45), and regarding the distribution by gender, 76.95% were women (n = 187).

In the second phase of the recruitment procedure, 2.2% of the Hungarian students of the University of Debrecen (from a total of 21.595 students) participated in our research. Thus, the second sample consisted of 474 persons, of whom 58 were identified as maladaptive daydreamers (12.24%). The youngest respondent was 18 and the oldest was 58 (M = 26.06; SD = 8.55). The gender distribution of this sample seemed to be similar to the first sample, as women represented 73.84% of the participants (n = 350).

As the aim of the study was to examine the differences between maladaptive and normal daydreamers, we combined the online community sample and the university student sample. The overall sample consisted of 717 persons, including 106 individuals who were screened as maladaptive daydreamers (14.78%). A chi-square test showed no significant differences regarding the gender distribution of the two groups, while an Independent Sample T-test revealed significant differences regarding the age (t(715) = 3.26, p = 0.0012), as maladaptive daydreamers seemed to be significantly younger. As for marital status (Fisher’s exact test, p < 0.001), education (Fisher’s exact test, p < 0.001) and employment status (χ2(4) = 25.04, p < 0.001), significant differences were found between the study groups. A remarkable finding of the study is that the majority of maladaptive daydreamers (55.66%) seemed to have single relationship status compared to the normal daydreamer group, where this rate was 34.21%. Another interesting result is that among maladaptive daydreamers the secondary education level was dominant (63.21%), whereas normal daydreamers seemed to have similar proportions of secondary (51.97%) and tertiary education (48.03%). Regarding the self-identified maladaptive daydreamer status, incorrect self-identification was relatively high among those who reported to be normal daydreamers, but who, based on the MDS-16-HU, were screened as maladaptive daydreamers (28.30%). 9.33% of the participants who reported to be maladaptive daydreamers, were screened as normal daydreamers. Table 1 shows the demographic characteristics of the two study groups.

**Table. 1:** Demographic characteristics of the study groups

	Maladaptive daydreamers (N = 106)	Normal daydreamers (N = 611)	Statistical tests	p value
Gender				
Male	26.42%	24.88%	χ^2^(1)= 0.1136	0.736
Female	73.58%	75.12%		
Age	26.33	30.13	t(715)=	=0.0012
(M, SD)	±8.71	± 11.46	3.2576	
Marital status				
Single	55.66%	34.21%	Fisher’s exact test	<0.001
In relationship/married	38.68%	60.07%		
Divorced	3.77%	5.4%		
Widowed	1.89%	0.33%		
Education				
Primary	2.83%	0%	Fisher’s exact test	<0.001
Secondary	63.21%	51.97%		
Tertiary	33.96%	48.03%		
Employment				
Student	57.55%	40.43%	χ^2^(4)= 25.0356	<0.001
Employed	20.75%	24.55%		
Student & employed	15.09%	32.08%		
Retired	0%	1.15%		
Other	6.6%	1.8%		
Self-identification				
Maladaptive	71.7%	9.33%	χ^2^(1) = 232.5634	<0.001
Normal	28.3%	90.67%		

#### Childhood Traumatic Experiences among Maladaptive and Normal Daydreamers

In order to compare maladaptive (n = 106) and normal daydreamers (n = 611) in terms of childhood traumatic experiences, we applied the Mann-Whitney U test. Based on the findings of previous studies (Abu-Rayya et al., 2020; Somer, 2002; Somer et al., 2019a; Somer & Herscu, 2017), we hypothesized that maladaptive daydreamers had experienced significantly higher level of trauma and/or more traumatic experiences compared to normal daydreamers.

As shown in Table 2, during the *first developmental period* (0–6 years), significant differences were found regarding the subscales of Neglect, Separation, Emotional abuse, Physical abuse, Sexual abuse, Witnessing and Other traumas. Maladaptive daydreamers reported significantly more severe and/or more frequent neglect experiences (p < 0.01), separation (p < 0.01), emotional abuse (p < 0.001), physical abuse (p < 0.001) and sexual abuse (p < 0.01). Furthermore, maladaptive daydreamers, during their first six years, were more likely to experience witnessing trauma (p < 0.001) and other types of traumatic experiences (p < 0.001). Regarding the subscale of Alcohol and drugs, no statistically significant difference was found between the groups. The median, Interquartile range (IQR), z-score and p value of the study groups for each trauma type are shown in Table 2.

**Table. 2:** Traumatic experiences during 0–6 years

	Normal daydreamers Median (Q25-Q75)	Maladaptive daydreamers Median (Q25-Q75)	z-score	p value
Neglect	0 (0–0.2)	0 (0–0.6)	−2.76	<0.01
Separation	0 (0–0.5)	0 (0–0.75)	−2.761	<0.01
Emotional abuse	0 (0–0.2)	0.2 (0–1)	−3.715	<0.001
Physical abuse	0 (0–0)	0 (0–0.33)	−5.238	<0.001
Sexual abuse	0 (0–0)	0 (0–0)	−3.177	<0.01
Witnessing	0 (0–0.17)	0.17 (0–0.5)	−4.699	<0.001
Other traumas	0 (0–0.17)	0.17 (0–0.5)	−3.368	<0.001
Alcohol and drugs	0 (0–0)	0 (0–0.5)	−1.68	0.0929

Regarding *the second developmental period* (7–12 years), maladaptive daydreamers scored significantly higher on the subscales of Neglect, Separation, Emotional, Physical and Sexual abuse, Witnessing trauma, and Other traumas. Findings suggest that maladaptive daydreamers, during their school years, experienced more frequent and/or more severe neglect (p < 0.001), separation (p < 0.05), emotional (p < 0.001), physical (p < 0.001), or sexual abuse (p < 0.001), became a witness (p < 0.001), or experienced other types of trauma (p < 0.001) compared to normal daydreamers. Regarding the subscale of Alcohol and drugs, no significant difference was found between the groups. The median, Interquartile range (IQR), z-score and p value of the study groups for each trauma type are shown in Table 3.

**Table. 3:** Traumatic experiences during 7–12 years

	Normal daydreamers Median (Q25-Q75)	Maladaptive daydreamers Median (Q25-Q75)	z-score	p value
Neglect	0 (0–0.4)	0.4 (0–1)	−3.887	<0.001
Separation	0.25 (0–0.75)	0.75 (0–1.25)	−2.369	<0.05
Emotional abuse	0.2 (0–0.6)	0.8 (0.2–1.8)	−5.824	<0.001
Physical abuse	0 (0–0.33)	0 (0–0.67)	−4.904	<0.001
Sexual abuse	0 (0–0)	0 (0–0)	−3.962	<0.001
Witnessing	0 (0–0.33)	0.25 (0–0.67)	−4.532	<0.001
Other traumas	0 (0–0.17)	0.17 (0–0.5)	−4.709	<0.001
Alcohol and drugs	0 (0–0.5)	0 (0–1)	−1.787	0.074

#### Dissociative Experiences among Maladaptive and Normal Daydreamers

To identify the relationship between maladaptive daydreaming and dissociative experiences, we compared the study groups’ score on the DISQ-H with the Mann-Whitney U test. Based on previous studies (Somer et al., 2016c; Somer & Herscu, 2017; Somer, 2018; Soffer-Dudek & Somer, 2018; Somer et al., 2019b), we presumed that maladaptive daydreamers possessed significantly higher levels of dissociative propensity than normal daydreamers, thus they would report more dissociative experiences.

Examining the total of DISQ-H score, we found a strong, significant difference between the two study groups (p < 0.001). Examining the subscales of DISQ-H further, significant differences were found between maladaptive and normal daydreamers on each dimension of dissociation. Maladaptive daydreamers compared to normal fantasizers reported more experiences of Identity confusion and fragmentation (p < 0.001), Loss of control (p < 0.001), Amnesia (p < 0.001) and Absorption (p < 0.05). The median, Interquartile range (IQR) and p value of the study groups for each dissociative subscale are shown in Table 4.

**Table. 4:** Presence of dissociative experiences among the two study samples

	Normal daydreamers Median (Q25-Q75)	Maladaptive daydreamers Median (Q25-Q75)	p value
Identity confusion and fragmentation	1.32(1.16–1.64)	2.32(1.8–2.96)	<0.001
Loss of control	1.89(1.5–2.33)	3.06(2.56–3.56)	<0.001
Amnesia	1.36(1.14–1.71)	1.96(1.57–2.5)	<0.001
Absorption	2.5(2–2.83)	2.67(2–3.17)	<0.05
Total DISQ-H score	1.57(1.37–1.86)	2.4(2.03–2.94)	<0.001

#### Examination of the Relationship between Childhood Trauma, Dissociative Experiences and Maladaptive Daydreaming

To identify the direct and indirect relationships of maladaptive daydreaming with traumatic childhood events and dissociative experiences, the Structural Equation Modelling (SEM) method was used. As an initial step, the univariate and multivariate normality of the data (i.e. traumatic experiences between the age of 0 and 6 years; dissociative experiences; maladaptive daydreaming) were tested with mvtest command. The results showed that each indicator violates the univariate normality assumption and all together violates the assumption of multivariate normality (Doornik-Hansen χ^2^(26) = 10,098.407, p < 0.001). We also tested the univariate and multivariate normality for the traumatic experiences in the second developmental period, dissociative experiences, and maladaptive daydreaming. The results revealed that these indicators also violate the univariate normality assumption and all together violates the assumption of multivariate normality (Doornik-Hansen χ^2^(26) = 11,385.201, p < 0.001).

Due to the non-normal distribution of the data, the Kruskal-Wallis Test was used to measure the influence of each trauma type on maladaptive daydreaming. Table 5 shows the results of the Kruskal-Wallis Test.

**Table. 5:** The influence of each trauma types (0–6 years) on maladaptive daydreaming

	χ2	df	p value
Neglect	33.089	12	<0.001
Separation	20.162	9	<0.05
Emotional abuse	33.689	14	<0.01
Physical abuse	35.74	7	<0.001
Sexual abuse	17.812	6	<0.01
Witnessing	41.193	12	<0.001
Other traumas	28.011	10	<0.01
Alcohol and drugs	7.944	5	0.1594

When comparing the traumatic experiences of the first developmental period, the results indicated that there was a statistically significant difference in the medians of MDS score between the following trauma types: Neglect (<0.001), Separation (<0.05), Emotional abuse (<0.01), Physical abuse (<0.001), Sexual abuse (<0.01), Witnessing (<0.001) and Other traumas (<0.01).

Table 6 shows the results of the Kruskal-Wallis Test regarding the influence of childhood traumatic experiences of the second developmental period on the MDS score.

**Table. 6:** The influence of each trauma types (7–12 years) on maladaptive daydreaming

	χ2	df	p value
Neglect	31.203	12	<0.01
Separation	19.155	11	0.0584
Emotional abuse	61.619	15	<0.001
Physical abuse	41.094	9	<0.001
Sexual abuse	8.756	8	0.3633
Witnessing	29.204	15	<0.05
Other traumas	53.011	10	<0.001
Alcohol and drugs	8.327	6	0.2151

The results revealed statistically significant difference in the medians of MDS score between the following trauma types of the second developmental period: Neglect (<0.01), Emotional abuse (<0.001), Physical abuse (<0.001), Witnessing (<0.05) and Other traumas (<0.001).

Then, the method of robust regression was applied to measure the direct impacts of the childhood traumatic experiences (which influence the MDS score based on the Kruskal-Wallis Test), the dissociative experiences, the gender, the age groups, and the level of education on maladaptive daydreaming. We tested the direct impacts of the variables on maladaptive daydreaming separately according to the two developmental periods of the traumatic experiences. Table 7 and Table 8 show the results of the analysis.

**Table. 7:** Direct impacts of childhood traumatic experiences (0–6 years), dissociative experiences, gender, age group, and level of education on maladaptive daydreaming (n = 379)

**Variable**	**Coefficient**	**T-value**	**p value**
Neglect	−2.952	−1.39	0.167
Emotional abuse	−2.396	−1.58	0.115
Separation	0.673	0.56	0.579
Physical abuse	2.243	1.07	0.287
Sexual abuse	3.868	1.17	0.241
Witnessing trauma	2.362	1.16	0.246
Other trauma	0.69	0.29	0.772
Identity confusion and fragmentation	11.936	6.39	<0.001
Loss of control	7.16	4.67	<0.001
Amnesia	−2.829	−1.79	0.075
Absorption	−0.735	−0.84	0.4
Gender	−0.113	−0.09	0.926
Age group	−0.257	−3.28	=0.001
Education	−0.668	−0.57	0.57

**Table. 8:** Direct impacts of childhood traumatic experiences (7–12 years), dissociative experiences, gender, age group, and level of education on maladaptive daydreaming (n = 549)

**Variable**	**Coefficient**	**T-value**	**p value**
Neglect	−2.202	−1.68	0.094
Emotional abuse	−1.261	−1.29	0.196
Physical abuse	2.427	1.84	0.066
Witnessing trauma	1.108	0.83	0.408
Other trauma	2.892	1.57	0.117
Identity confusion and fragmentation	9.389	6.47	<0.001
Loss of control	7.937	6.57	<0.001
Amnesia	−1.041	−0.79	0.429
Absorption	−0.05	−0.07	0.942
Gender	−0.423	−0.42	0.673
Age group	−0.254	−4.18	<0.001
Education	−0.052	−0.06	0.954

The results showed that seven trauma types of the first developmental period, the gender and the level of education did not exert direct effect on maladaptive daydreaming, while Identity confusion and fragmentation, Loss of control, and the age group had a significant direct effect on the MDS score.

The results revealed that five trauma types of the second developmental period, gender and the level of education did not exert direct effect on maladaptive daydreaming, while Identity confusion and fragmentation, Loss of control, and the age group had a direct effect on the phenomenon. These findings raise the possibility of mediation. The next step of the analysis aimed to reveal whether the dissociative experiences mediated the relationship between childhood trauma and maladaptive daydreaming. To test this hypothesis, Structural Equation Modeling was used by applying the Asymptotically Distribution-Free (ADF) estimation method. Table 9 presents the results of the first path analysis.

**Table. 9:** The results of the first path analysis (n = 379)

**Dependent variable**	**Independent variable**	**Coefficients**	**p value**
Maladaptive daydreaming	Identity confusion and fragmentation	11.538	<0.001
	Loss of control	6.54	=0.001
Identity confusion and fragmentation	Neglect	0.235	0.127
	Separation	0.012	0.836
	Emotional abuse	0.398	<0.001
	Physical abuse	0.249	0.1
	Sexual abuse	0.288	0.235
	Witnessing	−0.187	0.135
	Other traumas	0.116	0.402
Loss of control	Neglect	0.239	0.153
	Separation	0.06	0.423
	Emotional abuse	0.314	<0.001
	Physical abuse	0.281	0.108
	Sexual abuse	−0.101	0.698
	Witnessing	−0.17	0.238
	Other traumas	0.197	0.239

**Fig. 1:**
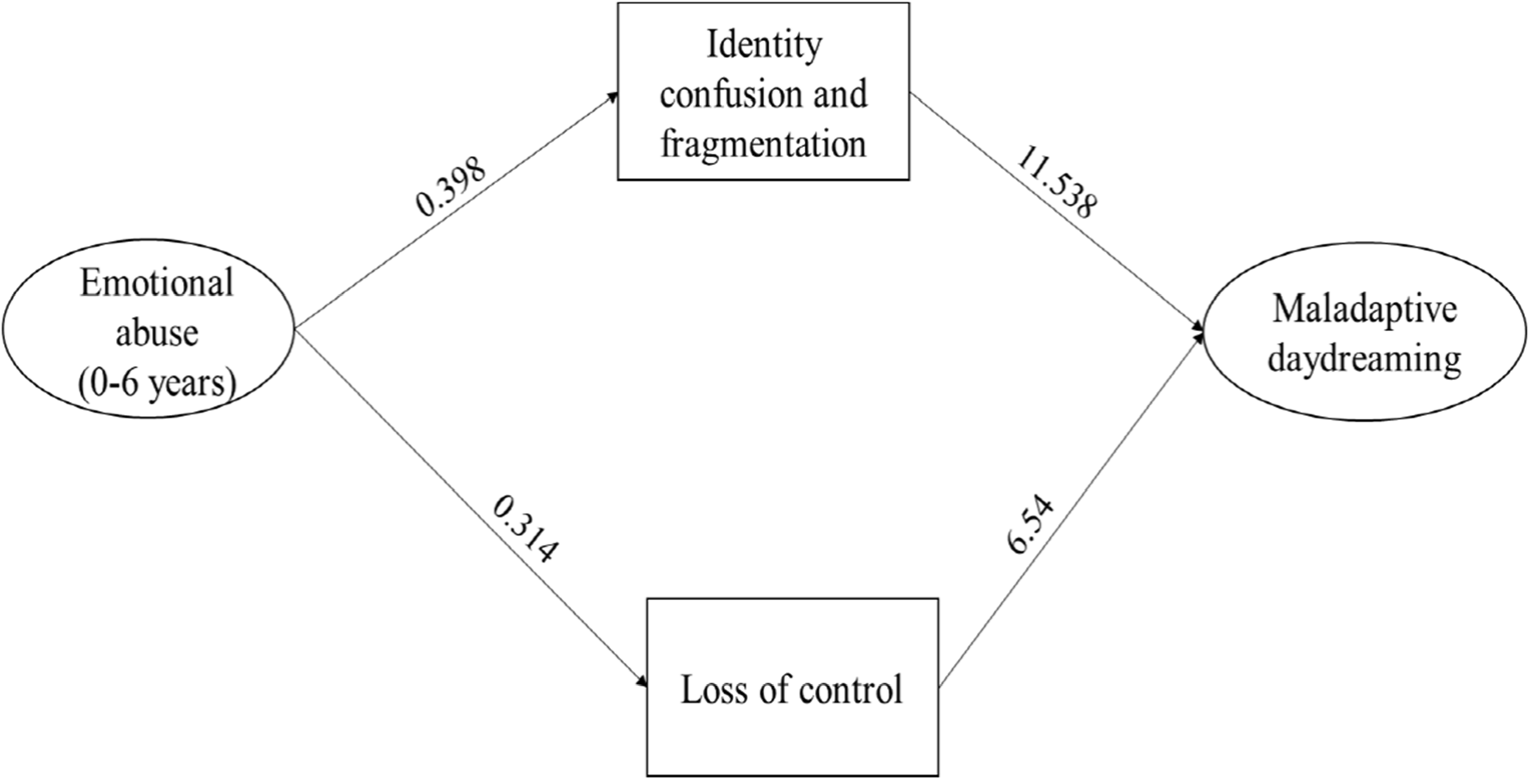
Path diagram presenting the significant associations between childhood trauma (0–6 years), dissociative experiences, and maladaptive daydreaming

The first model yielded an acceptable fit to the data (χ2(7) =8.038, p = 0.329; CFI = 0.99; TLI = 0.965; RMSEA = 0.020 [0.000–0.068], p = 0.806; SRMR = 0.101). The path diagram is presented in Fig. 1, which shows only the significant paths.

The first model revealed that Identity confusion and fragmentation had a significant direct effect (p < 0.001) on maladaptive daydreaming; Identity confusion and fragmentation was influenced by Emotional abuse (0–6 years) (p < 0.001). Loss of control over emotions, behaviours and thoughts also showed a strong, direct effect (p = 0.001) on maladaptive daydreaming. Of the specific trauma types, Emotional abuse (0–6 years) (p < 0.001) influenced the dimension of Loss of control.

Then, we also used the Structural equation Modeling to reveal the direct and indirect relationships between the variables. Table 10 presents the results of the second path analysis.

**Table. 10:** Results of the second path analysis (n = 549)

**Dependent variable**	**Independent variable**	**Coefficients**	**p value**
Maladaptive daydreaming	Identity confusion and fragmentation	10.009	<0.001
	Loss of control	7.735	< 0.001
Identity confusion and fragmentation	Neglect	0.139	0.152
	Emotional abuse	0.269	<0.001
	Physical abuse	0.07	0.497
	Witnessing	0.036	0.703
	Other traumas	0.289	0.012
Loss of control	Neglect	0.063	0.556
	Emotional abuse	0.366	<0.001
	Physical abuse	0.023	0.824
	Witnessing	0.013	0.905
	Other traumas	0.245	0.085

**Fig. 2:**
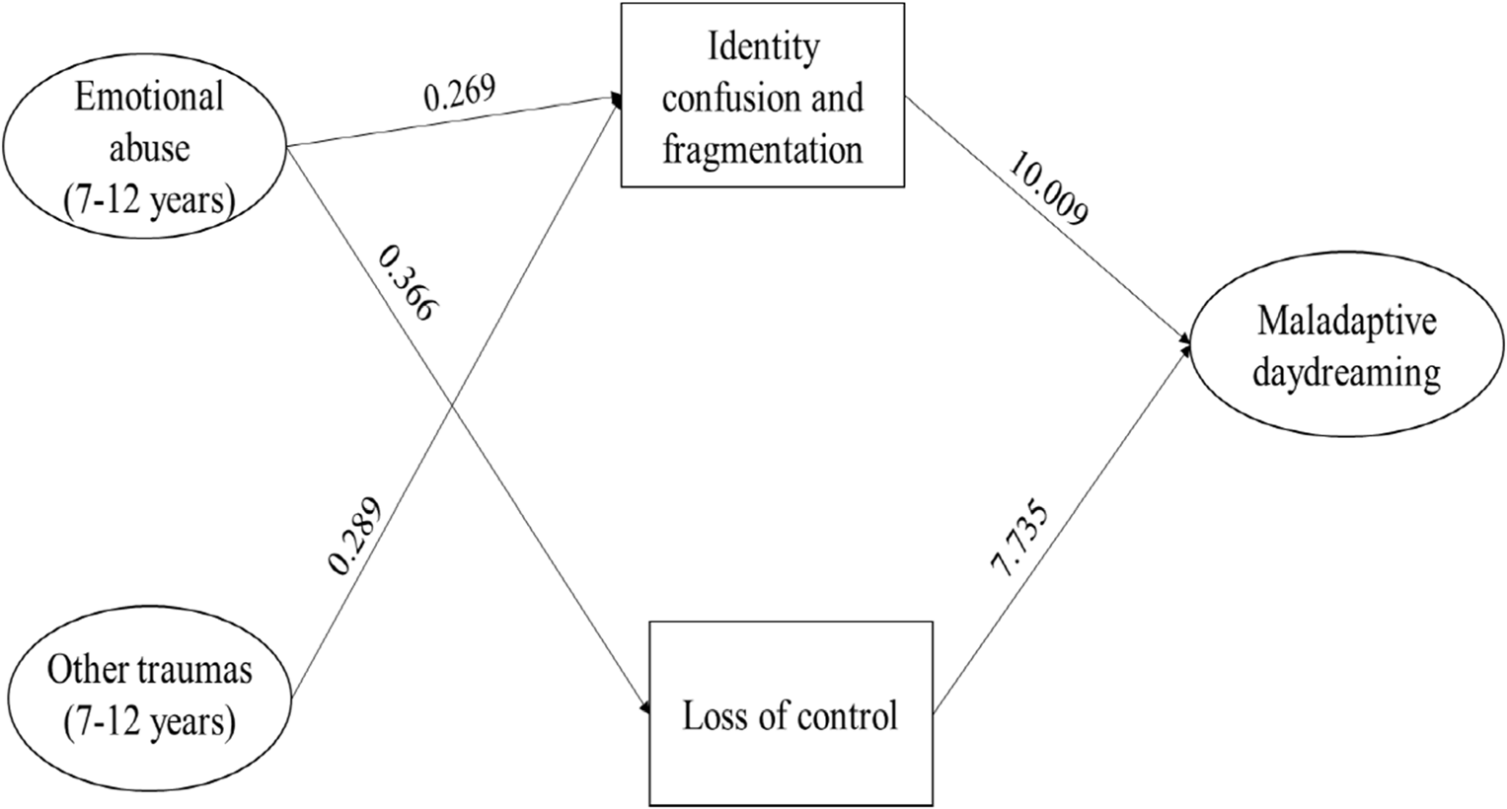
Path diagram presenting the significant associations between childhood trauma (7–12 years), dissociative experiences, and maladaptive daydreaming

The second model also yielded an acceptable fit to the data (χ2(5) =9.973, p = 0.076; CFI = 0.971; TLI = 0.897; RMSEA = 0.043 [0.000–0.081], p = 0.566; SRMR = 0.025). The second path diagram is presented in Fig. 2.

The second model revealed that Identity confusion and fragmentation had a significant direct effect (p < 0.001) on maladaptive daydreaming; Identity confusion and fragmentation was influenced by Emotional abuse (7–12 years) (p < 0.001) and Other traumas (7–12 years) (p = 0.012). Loss of control over emotions, behaviours and thoughts also showed a strong, direct effect (p < 0.001) on maladaptive daydreaming. Of the specific trauma types, Emotional abuse (7–12 years) (p < 0.001) influenced the dimension of Loss of control.

## Discussion

In our study, we aimed to examine specific segments of the etiology of maladaptive daydreaming, focusing on the potential trauma origin of the disorder and maladaptive daydreamers’ dissociative propensity.

Although since the publication of the seminal paper on maladaptive daydreaming (Somer, 2002) a core issue has been the potential effect of childhood traumatic experiences on the development of this phenomenon, the relationship between maladaptive daydreaming and childhood trauma has remained unclear. Our research provides evidence supporting previous findings suggesting the importance of traumatic experiences in the etiology of problematic daydreaming (Abu-Rayya et al., 2020; Somer, 2002; Somer et al., 2019a; Somer & Herscu, 2017; Somer et al., 2016a). The results of the Traumatic Antecedents Questionnaire indicated that, as regards the first developmental period (0–6 years), seven of the eight trauma types were significantly more prevalent among maladaptive daydreamers compared to normal daydreamers (except for Alcohol and drugs). A significant difference was found on the *Neglect* subscale suggesting that maladaptive daydreamers experienced more frequent and/or more severe neglect (such as lack of care; negligence; neglect because of familial problems). A significant difference on the subscale of *Separation* indicates that maladaptive daydreamers reported significantly more severe and/or more frequent separation experiences: separation from or divorce of parents; living with different people at different times; death or hospitalization of a significant person. A strong significant difference on the *Emotional abuse* subscale suggests that maladaptive daydreamers, compared to normal daydreamers, suffered from more intense emotional adversities, uncomfortable experiences, insults, inconsistent family rules and unfair punishments during early childhood. Study groups differed significantly regarding *Physical abuse,* as problematic daydreamers reported more frequent and/or more severe violent experiences (such as attacks, punches, or potential danger of physical injury or death). Maladaptive daydreamers also reported more frequent and/or more severe experiences of *Sexual abuse* during their early childhood, as they experienced more severe sexual harassments or sexual intercourse against their will, compared to normal daydreamers. The findings regarding the *Witnessing* subscale showed that maladaptive daydreamers, during their first six years, were more likely to witness physical violence, parental fights and out of control behaviours, events or scenes of a sexual nature, or even dead bodies. Maladaptive daydreamers scored significantly higher on the subscale of *Other traumas*; they were more likely to experience early childhood experiences such as hospitalization, a serious accident, a natural disaster or something terrible which remained a mystery to them.

In the second developmental phase (7–12 years) maladaptive daydreamers continued to experience significantly more frequent and/or more severe traumas, again except for Alcohol and Drugs in the family. Our findings suggest that maladaptive daydreamers, during their school years, experienced more frequent and/or more severe neglect; furthermore, compared to normal daydreamers, maladaptive daydreamers tended to suffer to a greater degree from severe separation, emotional, physical, and sexual abuse, and to be more likely to be witnesses, or to experience other types of trauma. The findings related to the second developmental period revealed the same tendency as in the first period.

These results are in line with the previously described maladaptive daydreaming model (Somer et al., 2016a). According to our results, a substantial proportion of individuals identified as maladaptive daydreamers in adulthood experienced significantly higher level of childhood trauma, and those who possess the innate capacity for vivid fantasy activity, might use their imaginary world to escape from the fearful, intolerable reality into the internal world, and to create safe and supporting relationships that were missing in their real life.

Previous studies have explored associations between dissociation and maladaptive daydreaming (Bigelsen et al., 2016; Soffer-Dudek & Somer, 2018; Somer & Herscu, 2017; Somer et al., 2017a; Somer et al., 2016c; Somer et al., 2019b). These findings were supported by the significant results on every subscale of the Hungarian version of the Dissociation Questionnaire. The present study highlighted that the dissociative propensity is typical of maladaptive daydreamers, as maladaptive daydreamers reported a significantly higher overall DISQ-H score (median: 2.4) than normal fantasizers (median: 1.57). Maladaptive daydreamers scored significantly higher on each subscale of dissociative experiences compared to normal daydreamers.

The findings related to the subscale of *Identity confusion and fragmentation* suggest that excessive daydreamers are more prone to experience depersonalization, derealization, and alternation of dissociated alter personalities without conscious control. Moreover, our results explored the idea that in our sample problematic daydreamers seemed to be less able to control their behaviours, emotions and thoughts, suggesting that their self-agency might be impaired. Individuals who score significantly higher on the scale of *Loss of control* also seem to be more prone to impulsivity (Vanderlinden et al., 1993). Maladaptive daydreamers, based on our results, also struggle with *Amnesia*, indicating that they have a lower level of ability to recall past events, and their past acts. Although the difference between the two groups on the subscale of *Absorption* was significant, the degree of significance was lower compared to the other three subscales. This finding suggests that maladaptive daydreamers experience increased awareness of their acts (such as eating, walking), a self-monitoring tendency, as well as a vivid evocation of the past as if it were the present. These results confirmed previous research findings (Bigelsen et al., 2016; Somer & Herscu, 2017), as maladaptive daydreamers in our sample were also more prone to absorption, i.e. to total attentional involvement.

The overall data provided by our research confirmed the findings of Somer and his colleagues (2016), which suggested that maladaptive daydreamers tend to distance themselves from external reality and events, just as they do from their bodies and mental states, while memory deficits are also prevalent. According to our findings, maladaptive daydreamers showed the highest scores on the Loss of control subscale. The following highest scores were measured on Absorption, Identity confusion and Amnesia subscales (in order), while the difference between maladaptive and normal daydreamers regarding Absorption scores proved to be the weakest.

Examining the direct effects of childhood trauma, dissociative experiences, gender, age groups and level of education, the results revealed that, regarding the first developmental period, none of the examined trauma types had a direct effect on maladaptive daydreaming. Other variables, namely Identity confusion and fragmentation and Loss of control had a positive direct effect on maladaptive daydreaming, while the age groups had a significant negative effect on maladaptive daydreaming, suggesting that this excessive form of fantasizing may decline with age. Regarding the second developmental period, the same tendency can be observed: Identity confusion and fragmentation, Loss of control and age had a significant direct impact on maladaptive daydreaming. These findings raised the possibility that dissociation mediates the relationship between childhood trauma and maladaptive daydreaming.

To assess the complex relationships between the variables examined, the Structural Equation Modeling was used. Our findings showed that, regarding the *first developmental period*, Emotional abuse significantly influenced dissociative experiences, i.e. Identity confusion and fragmentation and Loss of control, which significantly affected the phenomenon of maladaptive daydreaming. This result confirmed the findings of Somer and Herscu (2017), who provided evidence for the role of mediators (absorption and fantasy addiction) between trauma and problematic daydreaming. The results of the present study support the findings of Ferrante and her colleagues (2020), who revealed the mediating role of dissociation and shame between emotional trauma and maladaptive daydreaming. However, in our SEM model, only Identity confusion and fragmentation, and Loss of control exerted a direct effect on maladaptive daydreaming. Identity confusion and fragmentation was significantly influenced by emotional abuse. This suggests that emotional adversities, insults, inconsistent family rules, unfair treatment of the child, unachievable parental expectations and the consequential constant sense of failure might lead to depersonalization, derealization, and also to the presence of dissociated alter-personalities, which might turn the attention away from the external world towards the internal fantasy world, strengthening the tendency for excessive daydreaming. Loss of control seemed to be an influential factor in problematic daydreaming, also determined by the effect of emotional abuse. Our findings showed that the self-agency of the child, and the sense of control over behaviour, emotions and thoughts might be impaired by the intense emotional adversity of the child, and by the uncertainty and unpredictability of the childhood environment. Maladaptive daydreamers might find their sense of control in their internal world.

Regarding the *second developmental period*, Identity confusion and fragmentation and Loss of control had a significant effect on maladaptive daydreaming. The experience of Identity confusion and fragmentation was influenced by Emotional abuse and Other traumas, suggesting that insulting, unfairly punishing the child, or other types of (impersonal) trauma (such as serious illness, accident or hospitalization of the child, experiencing natural disaster, being notified by the serious injury or death of a close person, or experiencing other types of frightening or traumatic events) might lead to the fragmentation of identity, or experiences of depersonalization, derealization. Loss of control was affected by Emotional abuse, which corresponds to the results obtained for the first developmental period.

The two estimated SEM models presented in this study showed that emotional abuse during childhood can entail serious consequences for children’s development. During school years, apart from emotional abuse, traumatic experiences such as neglect, and other forms of trauma also might function as risk factors for dissociative experiences, which might directly influence the development of maladaptive daydreaming. Childhood maltreatment causes severe public health problems and a serious social-welfare problem in high-income countries, as every year 4–16% of children are physically abused and up to one in ten is neglected or psychologically abused. Five to 10 % of girls and up to 5% of boys experience contact sexual abuse during their childhood and the number who are exposed to some type of sexual abuse is up to three times higher (Gilbert et al., 2009). The early years of life are particularly important for the development of cognitive, socio-emotional, and physical abilities. Children show increased vulnerability to traumatic events, as substantial proportion of trauma experienced in childhood affects children under the age of 7 years: in the United States in 2008, more than 50% of children who were victims of abuse or neglect were between the ages of 0 and 7 years. As infants are not able to express their emotions verbally, the traumatic experience tends to affect the child’s behaviour: agitation, eating and sleeping disturbances may occur as a consequence. Preschoolers already have more tools to express their feelings, however, as they are characterized by egocentric thinking, they often attribute the cause of the events to themselves (Perlman & Doyle, 2012).

Early traumatic experiences have significant adverse effects on the development and growth, as well as they might undermine the positive and protective nature of attachment relationships (Perlman & Doyle, 2012). Furthermore, exposure to trauma in childhood can be related to short-term (e.g. sadness, anxiety, poor concentration) and long-term consequences (e.g. increased risk for poorer mental health and unfavourable health and behavioural outcomes) (Tobin, 2016). More specifically, child maltreatment is one of the causes of child morbidity and mortality, and has long-lasting consequences such as mental and physical health issues, alcohol and drug misuse, obesity, chronic diseases and health-risk behaviours (Gilbert et al., 2009). The academic literature indicates that emotional abuse has severe negative effects on children’s mental health (Norman et al., 2012). Norman et al. (2012) revealed that emotional abuse and neglect might contribute to the development of depressive and anxiety disorders, suicide attempts, drug misuse and high-risk sexual behaviour (Norman et al., 2012).

Child maltreatment is also associated to dissociative symptoms, and according to trauma theories, events that cause an extreme and immediate biological threat to the individual - who possesses limited resources of active defense strategies-, might lead to severe dissociative symptoms (Haferkamp et al., 2015). Childhood emotional maltreatment is the least obvious and therefore the least studied form of abuse, although presumably the most prevalent type of child maltreatment (O’Dougherty Wright et al., 2009). However, in the development of dissociative symptoms, apart from childhood sexual and physical abuse, studies confirmed the role of psychological forms of maltreatment, such as emotional abuse and neglect (Haferkamp et al., 2015; O’Dougherty Wright et al., 2009; Watson et al., 2006). A study (Haferkamp et al., 2015) also explored that emotional abuse is the strongest and most direct predictor of dissociation in a sample of traumatized women with a history of child abuse.

It can be presumed that individuals screened as maladaptive daydreamers were exposed to a significantly higher level of emotional abuse and other severely traumatic experiences during childhood compared to normal fantasizers. These early experiences might affect the developmental process of a coherent identity and the sense of control and self-agency. Those individuals who have an intense capacity for immersive daydreaming might use the fantasy world in a way to cope with the external and internal insecurities (Somer & Herscu, 2017). Previous studies explored that the coping strategy of finding shelter in the internal world initiates in childhood (Ferrante et al., 2020; Somer et al., 2016a). Ferrante and her colleagues (2020) also revealed that emotional trauma, although is not an essential condition for the development of maladaptive daydreaming, plays an important role in the severity of the phenomenon. Childhood emotional abuse and neglect form one of the potential pathways leading to maladaptive daydreaming, as “the child might excessively recur to dissociation” to cope with the adverse experiences, which might promote the absorption activity and impair the integration of mental experiences (Ferrante et al., 2020, p.9). Childhood trauma seems to be a potential predictor of maladaptive daydreaming if other risk factors are also present (such as dissociation, shame) (Ferrante et al., 2020). However, it also has to be mentioned that different developmental pathways might lead to maladaptive daydreaming. A substantial proportion of maladaptive daydreamers report no childhood traumatic experiences, but use their inborn capacity for daydreaming as a source of joy and creativity (Bigelsen & Schupak, 2011; Somer et al., 2016a). The pleasure found in the inner world itself - which has a rewarding and reinforcing impact - cannot fully explain the development and maintenance of problematic daydreaming. Researchers suggest that the addictive and compulsive nature of maladaptive daydreaming, the intense yearning for this mental activity and the impaired control over daydreaming might be key factors in understanding the phenomenon (Somer et al., 2016a). In clinical practice it might be important to explore the specific factors that preceed and maintain the phenomenon of maladaptive daydreaming (Ferrante et al., 2020). Results of the present study highlight the importance of exploring potential childhood traumatic experiences, especially the role of emotional abuse during the first 12 years of life, and the potential mediating role of dissociation in the etiology and maintenance of maladaptive daydreaming.

## Limitations

One major limitation of the study is the overrepresentation of female participants; therefore, these findings may not be generalizable to males. Another limitation is related to the online sampling method, which might further distort the sample composition. Our questionnaire package could be accessed primarily by the segments of the population who have an Internet connection and sufficient Information Technology skills to complete the questionnaire package. These factors might have influenced the educational level of the sample, as the majority of the participants had a secondary or tertiary education, while individuals with primary education were underrepresented. Future study objective would be the matching of the study groups on gender, age, and education to obtain a clearer delineation of the differences between the study groups. However, maladaptive daydreamers are difficult to recruit in healthcare institutions, psychiatric institutes, clinics or medical practices, and the disorder is surrounded by shame, guilt and secrets, as problematic daydreaming is hidden even from family members, friends and psychotherapists (Somer et al., 2016a). Even so, the reported willingness to participate in studies related to maladaptive daydreaming is significant (Somer et al., 2016b).

Another limitation of this study was that the methodology relied on self-report and on retrospective reporting of traumatic experiences. Assessing trauma experience retrospectively might be influenced by recall bias and the individuals’ subjective appraisal of past trauma events (Frissa et al., 2016). It should be noted that in the sample of maladaptive daydreamers, participants struggled with a higher level of amnesia than normal daydreamers, consequently, the recall of past events and acts (such as traumatic childhood experiences) might be impaired by failures in remembering.

It is also important to note that most studies in this field have been conducted on diverse international samples. A recent study (Soffer-Dudek et al., 2020) explored that those items of the MDS-16, which assess the urge to daydream and the extent to which this fantasy activity may be comforting, might have different meanings in different cultures. This finding might be explained by the fact that there are individual and cultural differences regarding the attitude toward daydreaming: in some cultures, this mental activity can be considered as a leisure activity, while in another culture daydreaming can be treated as a shameful aspect of life which should be hidden from other people. However, other aspects of maladaptive daydreaming (i.e. Impairment, Kinesthesia and Music) seemed to be invariant on a metric level, thus, these constructs are comprehended in the same way by the individuals across different cultures. To estimate the prevalence of maladaptive daydreaming and its cross-cultural invariance, representative studies in different countries should be carried out (Soffer-Dudek et al., 2020).

## Conclusions

With our findings we aimed to contribute to research into the etiology of maladaptive daydreaming, and to highlight the potential role of traumatic childhood experiences and dissociative propensity in the development of this mental disorder.

Among maladaptive daydreamers all trauma types occurred significantly more frequently compared to normal daydreamers, except Alcohol and drugs in the two developmental periods. The findings on the Dissociation Scale showed unequivocally that problematic daydreamers experienced significantly more dissociative experiences, involving the experience of identity alteration, loss of control over behaviours, emotions and thoughts, amnesiac symptoms and intense absorptive experiences. The present study also provided evidence that during early and later childhood suffering from trauma, particularly emotional abuse, might cause severe dissociative symptoms, which may directly lead to the development of maladaptive daydreaming.

One message of the study is to draw attention to the early detection of the disorder among the specialists of the field, and to provide useful data for the effective care and support of maladaptive daydreamers and for the development of psychotherapeutic interventions. During the therapeutic process an important step is the identification of the underlying causes of a disorder, which is essential for the appropriate therapeutic protocol. Based on our results, it seems justified to consider trauma origin and dissociative propensity as potential factors in the etiology of maladaptive daydreaming.

## Data Availability

The datasets generated during and/or analysed during the current study are available from the corresponding author on reasonable request.
